# Reciprocal Changes of Circulating Long Non-Coding RNAs *ZFAS1* and *CDR1AS* Predict Acute Myocardial Infarction

**DOI:** 10.1038/srep22384

**Published:** 2016-03-01

**Authors:** Ying Zhang, Lihua Sun, Lina Xuan, Zhenwei Pan, Kang Li, Shuangshuang Liu, Yuechao Huang, Xuyun Zhao, Lihua Huang, Zhiguo Wang, Yan Hou, Junnan Li, Ye Tian, Jiahui Yu, Hui Han, Yanhong Liu, Fei Gao, Yong Zhang, Shu Wang, Zhimin Du, Yanjie Lu, Baofeng Yang

**Affiliations:** 1 Department of Pharmacology, Harbin Medical University (the State-Province Key Laboratories of Biomedicine-Pharmaceutics of China, Key Laboratory of Cardiovascular Research, Ministry of Education), Harbin Medical University, Harbin, Heilongjiang, China; 2 Department of Epidemiology and Biostatistics, Public Health School, Harbin Medical University, Harbin, Heilongjiang, China; 3 Department of Cardiology, the First Affiliated Hospital, Harbin Medical University, Harbin, Heilongjiang, China; 4 Division of Pathophysiology (the State-Province Key Laboratories of Biomedicine-Pharmaceutics of China and the Key Laboratory of Cardiovascular Research, Ministry of Education), Harbin Medical University, Harbin, Heilongjiang, China; 5 Department of gerontology, the First Affiliated Hospital, Harbin Medical University, Harbin, Heilongjiang, China; 6 Laboratories of Medicine, the Second Affiliated Hospital, Harbin Medical University, Harbin, Heilongjiang, China; 7 Institute of Clinical Pharmacology, the Second Affiliated Hospital, Harbin Medical University, Harbin, Heilongjiang, China; 8 Department of Pharmacology and Therapeutics, Melbourne School of Biomedical Sciences, Faculty of Medicine, Dentistry and Health Sciences, University of Melbourne, Melbourne, Australia

## Abstract

This study sought to evaluate the potential of circulating long non-coding RNAs (lncRNAs) as biomarkers for acute myocardial infarction (AMI). We measured the circulating levels of 15 individual lncRNAs, known to be relevant to cardiovascular disease, using the whole blood samples collected from 103 AMI patients, 149 non-AMI subjects, and 95 healthy volunteers. We found that only two of them, Zinc finger antisense 1 (*ZFAS1*) and Cdr1 antisense (*CDR1AS*), showed significant differential expression between AMI patients and control subjects. Circulating level of *ZFAS1* was significantly lower in AMI (0.74 ± 0.07) than in non-AMI subjects (1.0 ± 0.05, *P* < 0.0001), whereas *CDR1AS* showed the opposite changes with its blood level markedly higher in AMI (2.18 ± 0.24) than in non-AMI subjects (1.0 ± 0.05, *P* < 0.0001). When comparison was made between AMI and non-AMI, the area under ROC curve was 0.664 for *ZFAS1* alone or 0.671 for *CDR1AS* alone, and 0.691 for *ZFAS1* and CDR1AS combination. Univariate and multivariate analyses identified these two lncRNAs as independent predictors for AMI. Similar changes of circulating *ZFAS1* and *CDR1AS* were consistently observed in an AMI mouse model. Reciprocal changes of circulating *ZFAS1* and *CDR1AS* independently predict AMI and may be considered novel biomarkers of AMI.

Acute myocardial infarction (AMI) is the worst threat to human lives and the quality of human life. Early detection of AMI with noninvasive and reliable biomarkers is the foremost step for minimizing ischemic damage to the myocardium. Clinically validated biomarkers like creatine kinase MB (CKMB) and cardiac troponin I (cTnI), currently considered as “gold standard” for AMI diagnosis^1^,^2^,^3^,^4^, have a number of pitfalls. Search for new biomarkers of AMI, particularly those for early diagnosis, is therefore a top-urgent mission and has actually been an endless effort from fundamental and clinical researchers worldwide.

In addition to protein biomarkers, recent studies have suggested the potential value of RNA biomarkers for AMI, e.g., microRNAs (miRNAs)^5^,^6^,^7^. More recently, long non-coding RNAs (lncRNAs), a new class of functional mRNA-like transcripts lacking significant open reading frames or protein-coding capacity^8^,^9^,^10^,^11^, are emerging as an important layer in the gene regulatory network. As opposed to miRNAs, another class of regulatory RNAs, that are only 19 ~ 25 nt long, lncRNAs range in length from 200 nts to ~100 kilobases (kb). The growing body of literature has provided ample evidence for the critical role of lncRNAs in controlling a wide spectrum of biological processes through diverse but yet poorly understood molecular mechanisms, despite that only a handful of lncRNAs have been functionally and molecularly characterized^10^,^11^,^12^. LncRNA expression is highly variable, with greater tissue specificity compared to protein-coding mRNAs, and only 1% of the lncRNAs are ubiquitously expressed across all tissues examined. These properties of lncRNAs make them potential new biomarkers for disease diagnosis and prognosis. Indeed, recent studies have unraveled that expression of lncRNAs is temporal- and spatial-dynamically regulated by many factors and aberrant expression of lncRNAs has been increasingly documented in developmental programs, cancers, neuronal disorders, diabetes, etc. Most prominently, lncRNAs are readily detectable in a number of human body fluids such as serum^13^, plasma^14^,^15^,^16^, saliva^17^, and urine^18^,^19^, making them promising and attractive in the search of novel biomarkers in body fluid samples and noninvasive and rapid diagnostic tool for disease diagnosis and prognosis.

On the basis of available data in the literature, we proposed that circulating lncRNAs are epigenetic biomarkers for AMI and can be used to predict cardiovascular risk. This study was designed to test our hypothesis by identifying circulating lncRNA biomarkers for AMI with human blood samples and in a mouse model with blood and myocardium tissues. We have also analyzed the power of candidate lncRNAs to predict cardiac risk event, correlated them with known biomarkers, and assessed the regulatory role of these lncRNAs in cardiac function.

## Results

### Clinical Characteristics of the Study Population

Blood samples were collected from a total of 138 AMI patients, 149 non-AMI control subjects, and 95 healthy volunteers. Among the 138 AMI blood samples, 103 were from patients with ischemic time ≤12 h (an average of ischemic time = 3.5 h), 20 with ischemic time ≤12 h but without complete medical records, and 15 with ischemic time ranging from 24–36 h. Therefore, only 103/138 AMI patients were included in our detailed statistical analyses in the following sections to comply with our goal for early detection, despite that the same experimental results held for true the patients with ischemic time longer than 12 h.

AMI patients were aged 60.71 ± 11.05 years, comparable with the healthy volunteers (HV, 54.30 ± 12.69 years) and non-AMI control subjects (57.85 ± 11.92 years). There were no significant differences between AMI and non-AMI in the status hypertension and diabetes mellitus (Table 1).

### Reciprocal Changes of *ZFAS1* and *CDR1AS* Blood Levels in AMI Patients

**Figure 1:**
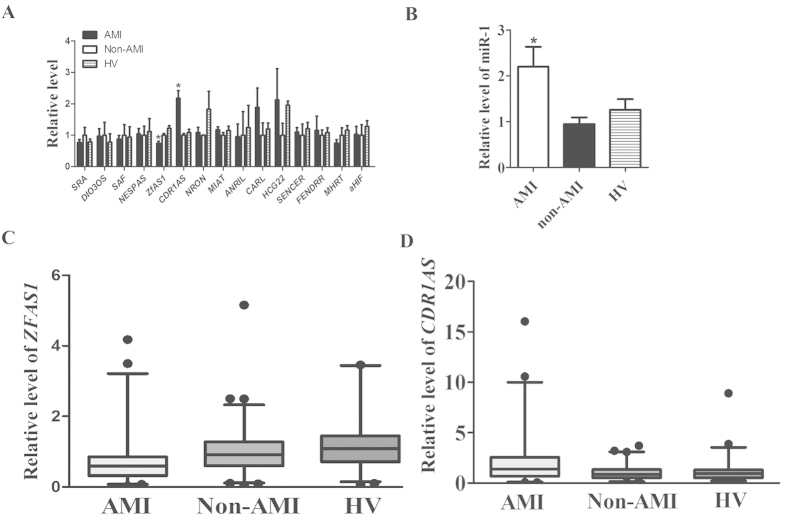
Changes of circulating lncRNAs levels in patients with acute myocardial infarction (AMI), with miR-1 as a positive control. (**A**) Circulating levels of lncRNAs were determined by quantitative real-time RT-PCR with the whole-blood samples from AMI patients, non-AMI control subjects, and healthy volunteers (HV). Only *ZFAS1* and *CDR1AS* demonstrated significant differences between AMI and healthy patients. *P* < 0.0001, n = 95 for HV and n = 149 for non-AMI control subjects. (**B**) Blood level of miR-1. *P* < 0.05, n = 30 for control and n = 32 for AMI. Data were present as means ± SEM in (**A,B**). (**C,D**) Box plot of blood *ZFAS1* and *CDR1AS* levels.

Our initial qPCR analysis included 15 then-known cardiac-specific or cardiac-related lncRNAs (http://cmbi.bjmu.edu.cn/lncrnadisease): *SRA*, *DIO3OS*, *SAF*, *NESPAS*, *MIAT*, *NRON*, *ANRIL*, *ZFAS1*, *CDR1AS*, *CARL*, *HCG22*, *SENCER*, *FENDRR*, *MHRT*, *aHIF*. As illustrated in Fig. 1A, of 15 lncRNAs tested, only *ZFAS1* (Zinc finger antisense 1) and *CDR1AS* (Cdr1 antisense) demonstrated significant differences in the whole blood samples between AMI, non-AMI, and HV. Circulating level of *ZFAS1* was significantly lower in AMI (0.74 ± 0.07) than in non-AMI subjects (1.0 ± 0.05, *P* < 0.0001) and healthy volunteers (1.22 ± 0.08, *P* < 0.0001), whereas *CDR1AS* showed the opposite changes with its blood level markedly higher in AMI (2.18 ± 0.24) than in control populations (1.0 ± 0.05 in non-AMI subjects, 1.09 ± 0.10 in healthy volunteers, *P* < 0.0001). Specifically, circulating level of *ZFAS1* was 25.7% and 39.2% lower in AMI than in non-AMI subjects and in volunteers (Fig. 1C, Table 2), whereas *CDR1AS* showed the opposite changes in the bloodstream with its level 2.2 fold and 2.0 fold higher in AMI than in non-AMI and in healthy subjects (Fig. 1D, Table 2). The median Ct value for *ZFAS1* was 23.3, ranging from 20.2–26.2; and the median Ct value for *CDR1AS* was 22.2 with a range from 17.9–26.6.

Since circulating miR-1, a miRNA belonging to another class of non-coding RNAs, has been documented to be a new miRNA biomarker for AMI by several laboratories^7^,^20^,^21^,^22^,^23^,^24^, we included this miRNA in our analysis for comparison. As depicted in Fig. 1B, miR-1 level was found to be significantly elevated in AMI blood samples, verifying the previous finding and serving as a positive control in this study. These results indicate that *ZFAS1* and *CDR1AS* both exist in the human blood with good stability and high abundance just like miR-1 (known as a highly stable and abundant miRNA species in the circulation).

### Evaluation of Circulating *ZFAS1* and *CDR1AS* as New Biomarkers for AMI

**Figure 2:**
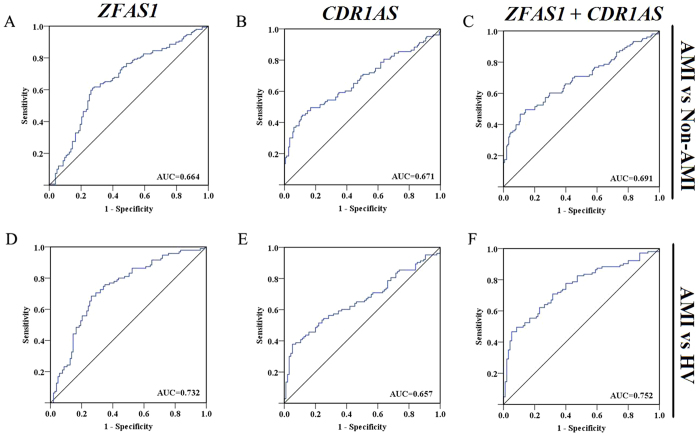
Receiver–operator characteristic (ROC) analysis of circulating *ZFAS1* or *CDR1AS* alone and in combination for predicting AMI. The area under ROC curve (AUC) was determined to evaluate the predictive power of circulating lncRNA levels for AMI using non-AMI subjects (**A–C**) or healthy volunteers (HV; **D–F**) as control. *ZFAS1* + *CDR1AS* indicates combination of the two lncRNAs.

Having established that *ZFAS1* and *CDR1AS* are present in the peripheral circulation and their blood levels are anomaly altered in AMI patients, we sought to determine the potential utility of circulating *ZFAS1* and *CDR1AS* as diagnostic biomarkers of AMI. To this end, ROC analysis was performed to evaluate the predictive power of circulating *ZFAS1* or *CDR1AS* alone and combination of the two for AMI. When comparison was made between AMI and non-AMI, the area under ROC curve (AUC) was 0.664 (95%CI = 0.594 ~ 0.733) for *ZFAS1* alone (Fig. 2A), 0.671 (95%CI = 0.600 ~ 0.742) for *CDR1AS* alone (Fig. 2B), and 0.691 (95%CI = 0.622 ~ 0.760) for *ZFAS1* and *CDR1AS* combination (*ZFAS1* + *CDR1AS*, Fig. 2C). When comparison was made between AMI and HV, the values were 0.732 (95%CI: 0.662 ~ 0.803), 0.657 (0.581 ~ 0.734), and 0.752 (0.684 ~ 0.820) for *ZFAS1*, *CDR1AS*, and *ZFAS1* + *CDR1AS*, respectively (Fig. 2D–F).

The univariate analysis with logistic regression showed that *ZFAS1*and *CDR1AS* were both predictors for AMI using either non-AMI (Table 3) or HV (Table 5) as control. The multivariate logistic regression analysis further identified *ZFAS1* and *CDR1AS* as an independent predictor for AMI (Tables 3b and 3d). It is noted that the odd ratio (OR), a measure of association between circulating *ZFAS1* level and AMI, for univariate analysis was <1 (0.512, 95% CI: 0.324 ~ 0.809, *P* value = 0.0042; Table 3) whereas the OR value for multivariate analysis was >1 (2.807, 95% CI: 0.559 ~ 14.086, *P* value = 0.2098; Table 4). The difference was ascribed to the inclusion of CHOL (circulating cholesterol level) in the multivariate analysis. Despite the difference, the fact that the OR values ≠ 1 (either <1 or >1 indicates the existence of an association between circulating *ZFAS1* and AMI. Particularly, the fact that *P* values for both univariate and multivariate analyses were <0.05 (Tables 3a and 3b) strongly support the view about the association between circulating *ZFAS1* and AMI. The univariate analysis showed that the odds ratios (OR) were 1.831 (95% CI: 1.425 ~ 2.352) for *CDR1AS* (*P* < 0.0001) between AMI and non-AMI (Table 3). The multivariate logistic regression analysis showed that the OR values were 3.989 (95% CI: 0.805 ~ 19.772 for *CDR1AS* (*P* = 0.0903) between AMI and non-AMI (Table 4). The univariate analysis showed that the OR values were 0.369 (95% CI: 0.227 ~ 0.597) for *ZFAS1* (*P* < 0.0001) and 1.535 (95% CI: 1.207 ~ 1.952) for *CDR1AS* (*P* < 0.0005) between AMI patients and healthy volunteers (Table 5). The multivariate logistic regression analysis showed that the OR values were 0.437 (95% CI: 0.186 ~ 1.028) for *ZFAS1* (*P* = 0.0578) and 2.186 (95% CI: 1.281 ~ 3.730 for *CDR1AS* (*P* = 0.0041) between AMI patients and healthy volunteers (Table 6).

### Relation of *ZFAS1* and *CDR1AS* to Conventional Prognostic Markers

In order to further evaluate the usefulness of circulating *ZFAS1* and *CDR1AS* as AMI biomarkers, we tested whether their levels correlated with cardiac risk factors, conventional AMI markers, and cardiac function parameters. The data summarized in Table 7 show that *ZFAS1* was negatively correlated with AST, LDH, and CK, HBDH, whereas *CDR1AS* was positively correlated with AST, LDH, and CK. Neither *ZFAS1* nor *CDR1AS* was correlated with diabetes mellitus, hypertension, smoking history, or cardiac contractile and electrophysiological functions.

### Reciprocal Changes of Circulating and Myocardial *ZFAS1* and *CDR1AS* Levels in a Mouse Model of AMI

**Figure 3:**
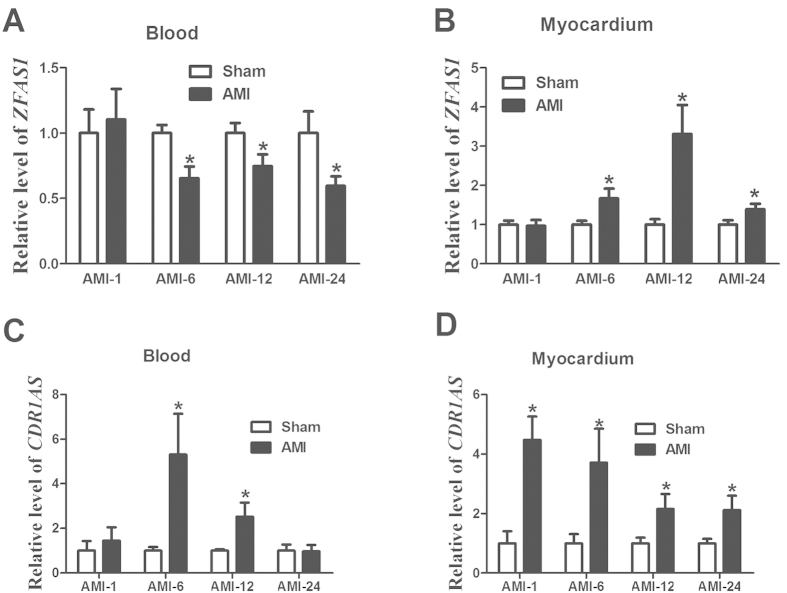
Changes of circulating (**A**,**C**) and myocardium (**B**,**D**) *ZFAS1* and *CDR1AS* levels in a mouse model of AMI at different time point (1 h, 6 h,12 h, and 24 h), determined by real-time RT-PCR methods. The number of blood samples ranged from 6 to 16 for different groups. AMI represent acute myocardial infarction with LAD. Data were present as means ± SEM.

Next, we sought to see if the alterations of circulating *ZFAS1* and *CDR1AS* in AMI patients could be reproduced in an experimental model of AMI with minimal confounding factors. RNA samples from whole blood and left ventricular myocardium of AMI mice and sham-operated control littermates were prepared at varying time points (1 h, 6 h, 12 h, 24 h). Real-time RT-PCR was carried out for changes of *ZFAS1* and *CDR1AS* in AMI samples relative to control samples. As illustrated in Fig. 3, the blood *ZFAS1* levels in AMI mice were decreased at all time points tested in our study and in contrast *CDR1AS* levels were increased, consistent with the findings in AMI patients. Notably, statistically significant decreases in circulating *ZFAS1* began as early as from 6 hour following LAD ligation, and similarly, significant elevation of *CDR1AS* occurred from 6 h after AMI.

Intriguingly, the changes of the myocardial expression of *ZFAS1* demonstrated the opposite direction to its circulating levels: the myocardium *ZFAS1* level was markedly increased in AMI at all four time points examined (Fig. 3). It is noted that the increases did not reach a statistical significant level until 6 h after AMI which lagged far behind its changes in the bloodstream. By comparison, the abundance of the myocardial *CDR1AS* expression followed the same direction of changes as its blood concentration, being upregulated in AMI relative to sham-operated control samples from 1 h after AMI (Fig. 3).

## Discussion

### Main Findings of the Study

We present here a study using whole blood samples collected from AMI patients at an average ischemic time of 3.5 h (~200 minutes) for early detection of circulating lncRNAs as potential biomarkers for AMI, by detecting the levels of 15 lncRNAs that are known to be relevant to cardiac development and cardiovascular disease. There a number of new findings in this study. (1) Of 15 known cardiac-relevant lncRNAs examined, *ZFAS1* and *CDR1AS* are the only two that demonstrated significant differences in their expressions between AMI patients and healthy subjects, as well as between experimental AMI and sham-operated mice. Blood *ZFAS1* and *CDR1AS* show reciprocal changes with *ZFAS1* being decreased and *CDR1AS* increased in the settings of AMI. (2) Significant changes of *ZFAS1* and *CDR1AS* in the bloodstream were detected at an average ischemic time of 3.5 h. (3) While either *ZFAS1* or *CDR1AS* was found to be well correlated with AMI, the combination of the two or the reciprocal changes of the two gives higher power of sensitivity and specificity of prediction, representing the superior biomarker for AMI. (4) *ZFAS1* and *CDR1AS* levels in the myocardium of AMI mice were both remarkably upregulated. Our findings indicate that circulating *ZFAS1* and *CDR1AS* are predictors of AMI and expression deregulation of lncRNAs may be a new molecular mechanism for cardiac disorders.

### Previous Studies on Circulating LncRNAs and Cardiovascular Disease

LncRNAs are newly discovered class of gene expression regulators. These long non-coding RNAs have garnered tremendous research interest worldwide, not only because they have been shown to participate in a wide spectrum of biological processes but also because they have been characterized as potential biomarkers for human disease. Indeed, lncRNAs have been documented to be high stable and readily detectable in a number of human body fluids such as serum^13^, plasma^14^,^15^,^16^, saliva^17^, and urine^18^,^19^. These properties of lncRNAs make them promising and attractive in the search of novel biomarkers in body fluid samples and noninvasive and rapid diagnostic tool for disease diagnosis and prognosis^25^,^26^.

During the course of the present study, a study on circulating lncRNAs as biomarkers for AMI was published^27^. This study compared the expression levels of 5 lncRNAs: *aHIF*, *ANRIL*, *KCNQLOT1*, *MIAT* and *MALAT1*. The levels of *aHIF*, *KCNQLOT1* and *MALAT1* were found increased in AMI relative to HV, whereas *ANRIL* level was decreased in AMI patients. In the present study, we focused our analysis on a selected set of lncRNAs (*SRA*, *DIO3OS*, *SAF*, *NESPAS*, *MIAT*, *NRON*, *ANRIL*, *ZFAS1*, *CDR1AS*, *CARL*, *HCG22*, *SENCER*, *FENDRR*, *MHRT*, *aHIF*) according to their cardiac-specific or cardiac-enriched expression using 103 blood samples from AMI patients, 149 samples from non-AMI control subjects, and 95 samples from healthy volunteers, in conjunction with blood samples from AMI mice. Among these lncRNAs, only *ZFAS1* and *CDR1AS* were found significantly altered with *ZFAS1* decreased whereas *CDR1AS* increased in their circulating levels in both human and mouse samples. The changes of circulating *ZFAS1* and *CDR1AS* levels in a mouse model of AMI at 12 hours can be detected with confidence after AMI. By comparison, among the lncRNAs selected for examination only two are overlapped between our study and the study by Vausort *et al.*: *MIAT* and *ANRIL*. We did not find any statistical significant or biologically meaningful difference of *MIAT* levels between AMI and healthy subjects, which is consistent with the finding in the study by Vausort *et al.* On the other hand, Vausort *et al.* reported lower levels of *ANRIL* in AMI patients, but our study failed to identify any significant changes of blood *ANRIL* level in AMI. The discrepancy could be explained by the difference of timing for blood sample collection: in the study by Vausort *et al.* the blood samples were harvested at the time of reperfusion with an average of 5 h after chest pain onset while in our study the samples were collected at an average ischemic time of 3.5 h for early detection of lncRNAs for AMI. The difference in the ischemic time may give rise to different levels of a given lncRNA as Vausort *et al.* found that lncRNA levels are dynamically regulated after MI. An alternative explanation would be the different samples used for lncRNA detection: Vausort *et al.* used leukocytes while we employed whole blood samples. Furthermore, under our experimental conditions, *ANRIL* level in bloodstream was extremely low with PCR cycle number over 32 which was virtually null. Another possible explanation is that the patients enrolled into the study by Vausort *et al.* were taking a variety of preadmission medications, whereas the patients included in our study were all on the first visit to our hospitals in emergency due to their chest pain and subsequent diagnosis of AMI prior to any medications. Thus, our results were presumably unaffected by medications.

*ZFAS1*, zinc finger antisense 1, is a transcript antisense to the 5’ end of the protein-coding gene Znfx1 and intronically hosts three previously undescribed C/D box snoRNAs (SNORDs): Snord12, Snord12b, and Snord12c. *ZFAS1* was found extremely stable with a half-life of >32 hrs in neuroblastoma cells^28^. *ZFAS1* is highly expressed in the mammary gland and is down-regulated in breast tumors compared to normal tissue. *CDR1AS* is an antisense to the cerebellar degeneration-related protein 1, belonging to the class of circular RNAs^29^,^30^. It is highly expressed in human brain, spinal cord, heart, lung, thymus and thyroid. In our samples, both *ZFAS1* and *CDR1AS* are fairly abundant RNA species in myocardium and blood as well, according to the Ct values from qPCR assays. Either of these two lncRNAs was found to be a good predictor of AMI though the changes of their levels went the opposite directions in blood, and combination of the two increased the power of prediction.

### Potential Significance of the Study

The discovery of deregulated lncRNAs not only sheds light on a new layer of regulatory network of human diseases, but it also opens up new opportunities for using these molecules as diagnostic markers and therapeutic targets. Our data suggest that lncRNAs as biomarkers can offer a number of advantages. First, they are sufficiently stable and can be readily detected in blood samples. Second, they can be quantified by highly sensitive methods such as PCR. Third, changes of lncRNAs in the blood may reflect the underlying mechanisms for the disease under detection. Our findings thus add that expression profiling and quantification of the lncRNA complement of the cardiac transcriptome in the systemic circulation will conceivably provide new venues for early diagnosis and treatment of the heart disease.

### Possible Limitations of the Study

Our measurement was limited to only a subset of lncRNAs without global transcriptome profiling. The data acquired thus do not provide us the overall picture of lncRNA presentation in blood and myocardium of AMI patients, nor do they define the lncRNA signature in AMI patients. There is well a possibility that we have missed out other important lncRNA markers for AMI. Nonetheless, the lncRNAs selected for our study are those that have been shown to be able to cause cardiac disorders or are abundantly expressed in heart cells. Fortunately, we were able to identify from this small group two lncRNAs that offer reliable detection with high-sensitivity and reproducibility. There is a hope that using high-throughput methods would allow for identification of better lncRNA biomarkers for AMI in future studies.

Another limitation of the study is the unknown sources of *ZFAS1* and *CDR1AS* in the blood stream. The release of RNA into the blood is thought to be related to the apoptosis and necrosis of cells and/or is the result of secretion from cells. LncRNAs are detectable in the serum and plasma, being surprisingly stable in spite of the fact that high amounts of RNases circulate in the blood stream, implying that these molecules may be protected from degradation by its packaging into microparticles, such as exosomes, microvesicles, apoptotic bodies, and apoptotic microparticles^31^. The reported RNA content of microvesicles and exosomes thus far includes primarily small miRNAs and long protein-coding mRNAs^32^.

Finally, the control subjects (including both non-AMI and healthy populations) in our study were not examined for any AMI biomarkers. While such a criterion for inclusion of controls is expected to give rise to minimal confounding results, a concomitant limitation is the inability to compare with the established risk markers in our analyses. Nevertheless, comparison between our results and the published data on cTnI^33^, an established risk marker of AMI, indicates that *ZFAS1/CDR1AS* and cTnI share a similar predictive power as early markers for AMI. In our study, the area under ROC curve (AUC) between AMI and HV was 0.732, 0.657 and 0.752 for *ZFAS1*, *CDR1AS*, and *ZFAS1* + *CDR1AS*, respectively; it is nearly identical to the AUC of 0.74 for cTnI.

## Summary

Collectively, we detected 15 known cardiac - relevant lncRNAs for their levels in the blood samples of AMI patients, and found that only two of them, *ZFAS1* and *CDR1AS*, demonstrated significant differences in their circulating levels between AMI patients and control subjects. Circulating *ZFAS1* and *CDR1AS* showed opposite changes with *ZFAS1* being decreased and *CDR1AS* increased in AMI. Moreover, while either *ZFAS1* or *CDR1AS* was found to be well correlated with AMI, the combination of the two or the reciprocal changes of the two gives higher power of sensitivity and specificity of prediction, representing the superior biomarker for AMI. Our findings suggest circulating *ZFAS1* + *CDR1AS* as a new biomarker of AMI.

## Materials and Methods

### Participants

Between February 2013 and November 2014, 138 AMI patients and 95 healthy volunteers (HV) and 149 non-AMI control subjects presented to the First Affiliated Hospital and the Second Affiliated Hospital of Harbin Medical University (Harbin, China). AMI was previously described^34^,^35^: see supplementary methods. The healthy volunteers were recruited at the time of regular annual medical checkup and the non-AMI subjects were recruited from the patients who visited the First Affiliated Hospital and the Second Affiliated Hospital of Harbin Medical University with conditions other than AMI. The non-AMI subjects were recruited from the patients without AMI. All patients and control subjects included in our study belong to Han people (Han nationality). These patients were all on the first visit to our hospitals in emergency due to their chest pain and subsequent diagnosis of AMI prior to any medications to avoid their possible influence on study results. The clinical characteristics of the study population are summarized in Table S1.

### Ethical Approval of Studies and Informed Consent

All experimental protocols were approved by the Ethnic Committee for Use of Human Samples of the Harbin Medical University and the methods were carried out in accordance with the approved guidelines. All human investigators procedures were approved by the Institutional Research Board of Harbin Medical University (No. HMUIRB-20140026). For investigations of humans, written informed consent was obtained from the study participants and participants received a stipend.

All animal procedures were approved by the Institutional Animal Care and Use Committee at Harbin Medical University (No. HMUIRB-2008-06) and the Institute of Laboratory Animal Science of China (A5655-01).

### Collection and Handling of Human Blood Samples

For lncRNA detection, whole blood (WB) samples (1 mL per patient) were drawn from the study subjects via a direct venous puncture into tubes containing sodium citrate. For AMI, peripheral blood samples were collected within an average ischemic time of 3.5 h prior to blood draw.

### Quantitative Real-Time Reverse Transcription (RT)-Polymerase Chain Reaction (PCR)

Total RNA was isolated from 1 mL whole blood sample using phenol/chloroform extraction procedures and real-time RT-PCR was performed as described before^20^,^21^. The PCR primer pairs are listed in Table S2.

### Animals

C57BL/6 mice ranging from 10 weeks to 12 weeks in age and weighed between 25–30 g each were provided by the experimental animal Center of Harbin Medical University. All experimental protocols were approved by the Ethnic Committee for Use of Animals Samples of the Harbin Medical University and the methods were carried out in accordance with the approved guidelines. And all the experiments were conformed to the Guide for the Care and Use of Laboratory Animals published by the US National Institutes of Health (NIH Publication No. 85–23, revised 1996).

### Acute Myocardial Infarction Model (AMI)

AMI was induced by left anterior descending coronary artery (LAD) ligation, as described in our previous study^21^.

### Collection and Handling of Mouse Blood Samples

Blood samples were drawn directly from mouse hearts, and the hearts were then dissected post AMI 12 h. These samples were immediately used for total RNA isolation as previously described^20^.

### Statistical Analysis

Categorical data were presented with count and percentile. Continuous variables were described as means ± SD, min, max, median and interquartile range. The statistical analyses were described in detail in supplementary methods. All analyses were carried out with SAS 9.1 (Serial No. 989155) except that ROC was done with SPSS v17.0 software. The significant level was set at 0.05 and two-tailed *P* values < 0.05 were considered statistically significant.

## Additional Information

**How to cite this article**: Zhang, Y. *et al.* Reciprocal Changes of Circulating Long Non-Coding RNAs *ZFAS1* and *CDR1AS* Predict Acute Myocardial Infarction. *Sci. Rep.* **6**, 22384; doi: 10.1038/srep22384 (2016).

## Supplementary Material

Supplementary Information

## Figures and Tables

**Table 1:**
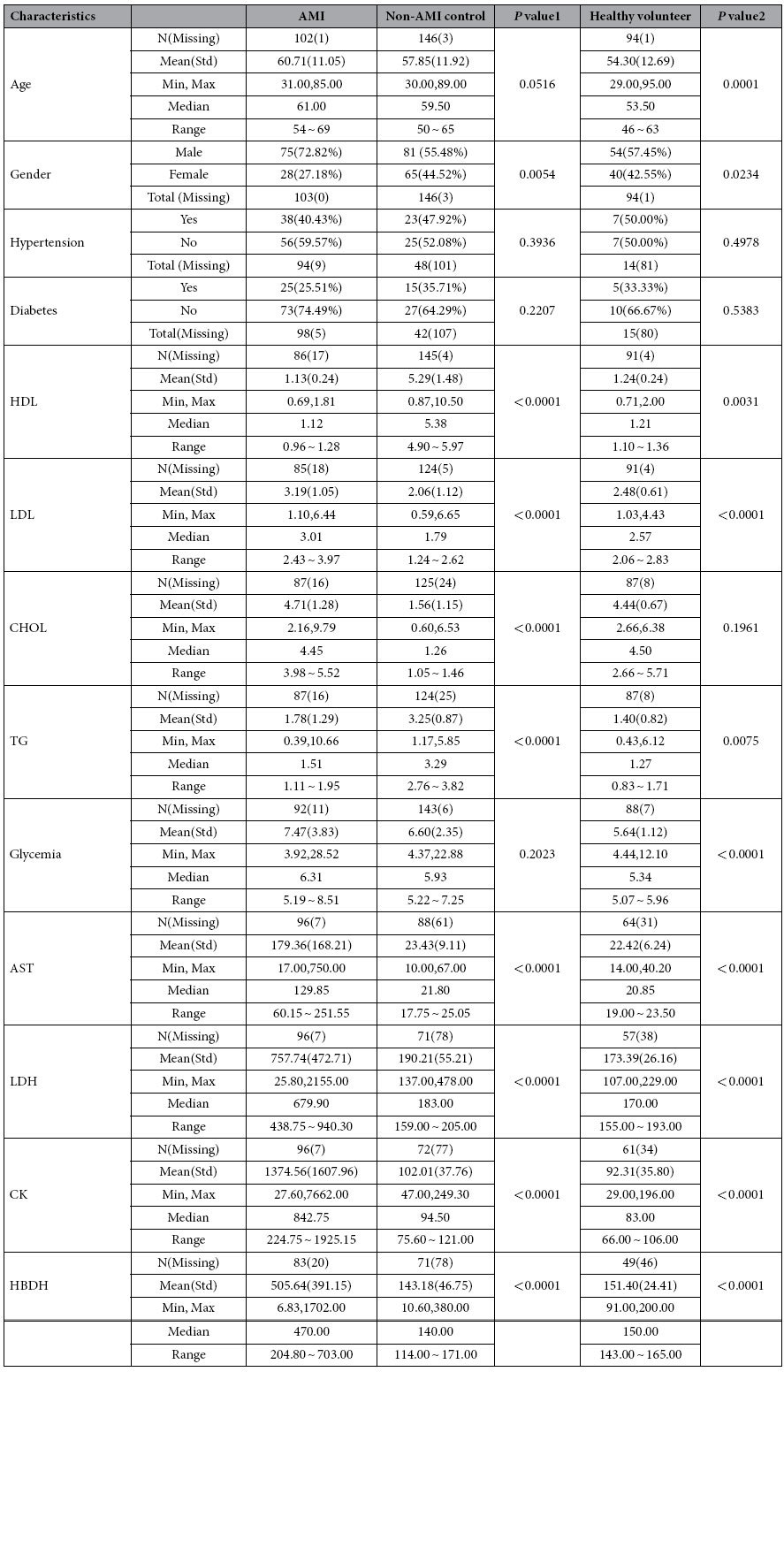
The demographic characteristics and AMI-relevant indicators in AMI patients, non-AMI control subjects and healthy volunteers.

AST: aspartate transaminase; CHOL: total cholesterol; CK: creatine kinase; HBDH: hydroxybutyrate dehydrogenase; HDL: high density cholesterol; LDH: lactic dehydrogenase; LDL: low density cholesterol.

**Table 2:**
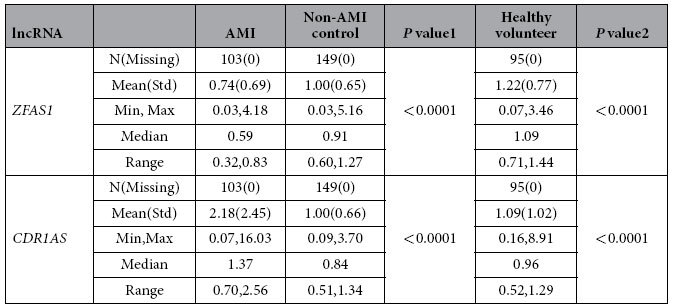
The Statistical Analysis of Circulating *ZFAS1* and *CDR1AS*.

*P* values 1 are for AMI *vs* Non-AMI, *P* values 2 are for AMI *vs* Healthy volunteer.

**Table 3:** Univariate regression analysis for the association of *ZFAS1* and *CDR1AS* with demographic characteristics between AMI patients and non-AMI control subject.

Parameter	Estimate	SE	Chi-Square	*P* value	OR	95%CI	
						lower	upper
*ZFAS1*	−0.6695	0.2337	8.2109	0.0042	0.512	0.324	0.809
*CDR1AS*	0.6046	0.1279	22.3575	<0.0001	1.831	1.425	2.352
Age	0.0215	0.0114	3.5890	0.0582	1.022	0.999	1.045
Gender	0.7651	0.2771	7.6238	0.0058	2.149	1.249	3.699
Blood sugar	0.0958	0.0473	4.0985	0.0429	1.101	1.003	1.207
HDL	1.4455	0.1718	70.8076	<0.0001	4.244	3.031	5.943
LDL	−1.5691	0.2116	55.0011	<0.0001	0.208	0.138	0.315
TG	0.8834	0.1444	37.4498	<0.0001	2.419	1.823	3.210
CHOL	−2.6585	0.7385	12.9578	0.0003	0.070	0.016	0.298

OR: odds ratio; CI: confidence interval.

**Table 4:** Multivariate regression analysis for the association of *ZFAS1* and *CDR1AS* with demographic characteristics between AMI patients and non-AMI control subjects.

Parameter	Estimate	SE	Chi-Square	*P* value	OR	95%CI	
						lower	upper
*ZFAS1*	1.0321	0.8230	1.5726	0.2098	2.807	0.559	14.086
*CDR1AS*	1.3835	0.8167	2.8694	0.0903	3.989	0.805	19.772
Age	−0.0512	0.0536	0.9122	0.3395	0.950	0.855	1.055
Gender	0.9524	1.0404	0.8380	0.3600	2.592	0.337	19.915
Blood sugar	0.0979	0.1911	0.2623	0.6085	1.103	0.758	1.604
HDL	−1.7646	0.9086	3.7717	0.0521	0.171	0.029	1.016
LDL	−0.1440	0.2734	0.2772	0.5985	0.866	0.507	1.480
TG	1.6917	0.9918	2.9093	0.0881	5.429	0.777	37.924
CHOL	−4.3830	1.3252	10.9388	0.0009	0.012	<0.001	0.168

OR: odds ratio; CI: confidence interval.

**Table 5:** Univariate regression analysis for the association of *ZFAS1* and *CDR1AS* with demographic characteristics between AMI patients and healthy volunteers.

Parameter	Estimate	SE	Chi-Square	*P*value	OR	95%CI	
						lower	upper
*ZFAS1*	−0.9980	0.2464	16.4092	<0.0001	0.369	0.227	0.597
*CDR1AS*	0.4283	0.1227	12.1781	0.0005	1.535	1.207	1.952
Age	0.0459	0.0129	12.5689	0.0004	1.047	1.021	1.074
Gender	0.6852	0.3042	5.0717	0.0243	1.984	1.093	3.602
Blood sugar	0.4420	0.1161	14.5044	0.0001	1.556	1.239	1.953
HDL	−1.9724	0.6868	8.2479	0.0041	0.139	0.036	0.535
LDL	1.0480	0.2207	22.5423	<0.0001	2.852	1.850	4.395
TG	0.4165	0.1967	4.4828	0.0342	1.517	1.031	2.230
CHOL	0.2641	0.1554	2.8875	0.0893	1.302	0.960	1.766

OR: odds ratio; CI: confidence interval.

**Table 6:** Multivariate regression analysis for the association of *ZFAS1* and *CDR1AS* with demographic characteristics between AMI patients and healthy volunteers.

Parameter	Estimate	SE	Chi-Square	*P* value	OR	95%CI	
						lower	upper
*ZFAS1*	−0.8270	0.4358	3.6001	0.0578	0.437	0.186	1.028
*CDR1AS*	0.7822	0.2726	8.2341	0.0041	2.186	1.281	3.730
Age	0.0499	0.0224	4.9911	0.0255	1.051	1.006	1.098
Gender	0.6747	0.6435	1.0996	0.2943	1.964	0.556	6.930
Blood sugar	0.2792	0.1484	3.5423	0.0598	1.322	0.989	1.768
HDL	2.1829	1.4824	2.1684	0.1409	8.872	0.486	162.108
LDL	4.4150	0.9635	20.9973	<0.0001	82.679	12.510	546.407
TG	1.1201	0.4272	6.8758	0.0087	3.065	1.327	7.080
CHOL	−3.6087	0.9621	14.0687	0.0002	0.027	0.004	0.179

OR: odds ratio; CI: confidence interval.

**Table 7:** Spearman’s rank correlation analysis for the association of *ZFAS1* and *CDR1AS* with cardiac risk factors, AMI biomarkers and cardiac function parameters in AMI patients.

	*ZFAS1*		*CDR1AS*	
	Coefficient	*P*	Coefficient	*P*
Cardiovascular risk factors				
Age	−0.14543	0.0071	0.00941	0.8624
Gender	−0.01964	0.7170	−0.27672	<0.0001
Diabetes	0.06353	0.4322	−0.07592	0.3478
Hypertension	0.05540	0.4921	0.04995	0.5358
Smoking	0.07969	0.4684	−0.14758	0.1777
HDL	0.07340	0.1889	−0.13414	0.0160
LDL	−0.01136	0.8396	0.09472	0.0907
CHOL	−0.02210	0.7035	0.15780	0.0063
TG	0.01888	0.7455	−0.11924	0.0397
Cardiac biomarkers				
cTnI	−0.08305	0.6201	−0.23340	0.1585
AST	−0.31188	<0.0001	0.22618	0.0003
LDH	−0.28928	<0.0001	0.28244	<0.0001
CK	−0.16315	0.0134	0.17946	0.0065
CKMB	−0.12912	0.1748	0.11498	0.2274
HBDH	−0.14003	0.0463	0.16458	0.0190
Cardiac function				
E/A	0.12059	0.2930	0.09910	0.3880
EF	0.06547	0.5375	−0.09906	0.3502
FS	−0.00606	0.9569	−0.06679	0.5510
Electrocardiogram				
QRS	0.00586	0.9613	−0.00245	0.9838
QT	−0.00074	0.9951	0.09224	0.4442
QTc	−0.01337	0.9119	−0.01778	0.8830
PR	0.00360	0.9784	−0.17636	0.1815
P	0.04044	0.7510	0.00693	0.9567
RR	−0.01216	0.9216	0.05646	0.6474
PP	0.12888	0.2949	0.03728	0.7628
P (^o^)	−0.14728	0.2494	−0.10992	0.3911
QRS (^o^)	−0.13512	0.2647	−0.02287	0.8509
T (^o^)	−0.10579	0.3906	−0.09140	0.4585
Heart rate	−0.01139	0.9232	−0.07593	0.5202
ST-segment elevation	−0.02231	0.8230	−0.00080	0.9936

HDL: high density cholesterol; LDL: low density cholesterol; CHOL: total cholesterol; TG: triglycerides; cTnI: cardiac troponin I; AST: aspartate transaminase; LDH: lactic dehydrogenase; CK: creatine kinase ; CKMB: isoenzyme creatine kinase; HBDH: hydroxybutyrate dehydrogenase; E/A: ratio of E velocity to A velocity; EF: ejection fraction; FS: left ventricular shortening fraction; QRS, QT, QTc, PR, P, RR, PP(ms): interval of QRS, QT, QTc, PR, P, RR, PP; P (^o^), QRS (^o^), T (^o^): axis for P, QRS and T.
