# Supplementary material for: Reciprocal Changes of Circulating Long Non-Coding RNAs ZFAS1 and CDR1AS Predict Acute Myocardial Infarction
Source: Sci Rep. 2016 Mar 1;6:22384. doi: 10.1038/srep22384 (PMC4772828; doi:10.1038/srep22384)
Supplement: Supplementary Information [file srep22384-s1.doc]

**Reciprocal Changes of Circulating Long Non-Coding RNAs *ZFAS1* and *CDR1AS* Predict Acute Myocardial Infarction**

Ying Zhang^1†^, Lihua Sun^1†^, Lina Xuan^1†^, Zhenwei Pan^1^, Kang Li^2^, Shuangshuang Liu^1^, Yuechao Huang^1^, Xuyun Zhao^1^, Lihua Huang^1^, Zhiguo Wang^1^, Yan Hou^2^, Junnan Li^2^, Ye Tian^3,4^, Jiahui Yu^3^, Hui Han^5^, Yanhong Liu^6^, Fei Gao^6^,Yong Zhang^1^, Shu Wang^3^, Zhimin Du^7^, Yanjie Lu^1^*, Baofeng Yang^1^*

^1^Department of Pharmacology, Harbin Medical University (the State-Province Key Laboratories of Biomedicine-Pharmaceutics of China, Key Laboratory of Cardiovascular Research, Ministry of Education), Harbin Medical University; ^2^Department of Epidemiology and Biostatistics, Public Health School, Harbin Medical University; ^3^Department of Cardiology, the First Affiliated Hospital, Harbin Medical University; ^4^Division of Pathophysiology (the State-Province Key Laboratories of Biomedicine-Pharmaceutics of China and the Key Laboratory of Cardiovascular Research, Ministry of Education), Harbin Medical University; ^5^Department of gerontology, the First Affiliated Hospital, Harbin Medical University; ^6^Laboratories of Medicine and ^7^Institute of Clinical Pharmacology, the Second Affiliated Hospital, Harbin Medical University, Harbin, Heilongjiang, China.

^†^Authors with equal contributions to this study.

*Corresponding

Bao-Feng Yang: e-mail: [yangbf@ems.hrbmu.edu.cn](mailto:yangbf@ems.hrbmu.edu.cn) or Yanjie Lu: yjlu2008@163.com;

Postal address: Department of Pharmacology (the State-Province Key Laboratories of Biomedicine-Pharmaceutics of China), Harbin Medical University, 157 Baojian Road, Nangang District, Harbin, Heilongjiang, China 150081;

Tel.: +86 451 8667-1354; Fax: +86 451 8667-1354.

**Supplementary Methods**

**Participants**

Between February 2013 and November 2014, 103 AMI patients and 95 healthy volunteers (HV) and 149 non-AMI control subjects presented to the First Affiliated Hospital and the Second Affiliated Hospital of Harbin Medical University (Harbin, China). AMI was diagnosed based on combination of several parameters: ischemic symptoms plus increased cardiac troponin I (cTnI) and creatine kinase-MB (CKMB), appearance of pathological Q wave, and ST-segment elevation or depression defined by the European Society of Cardiology/American College of Cardiology . Baseline ECG was recorded in all participants. Informed written consents were obtained from all participants and all investigations conformed to the principles of the Declaration of Helsinki. The study protocols were procured in accordance with the guidelines of and approved by the Ethics Committee of the Harbin Medical University. The clinical characteristics of the study population are summarized in **Table 1**.

**Collection and Handling of Human Blood Samples**

For lncRNA detection, whole blood (WB) samples (1 mL per patient) were drawn from the study subjects via a direct venous puncture into tubes containing sodium citrate. For AMI, peripheral blood samples were collected within an average ischemic time of 3.5 h prior to blood draw. The samples were used directly for RNA isolation.

**Quantitative Real-Time Reverse Transcription (RT)-Polymerase Chain Reaction (PCR)**

Total RNA was isolated from 1 mL whole blood sample using phenol/chloroform extraction procedures as described before. The first-strand cDNA was synthesized using the Reverse Transcription System (Promega) according to the manufacturer’s instructions as described previously^3^. The SYBR Green PCR Master Mix Kit (Applied Biosystems) was used in real-time PCR for relative quantification of lncRNAs. PCR was performed on 7500 FAST Real-Time PCR System (Applied Biosystems). The PCR primer pairs are listed in **Table S1**. Relative expression of lncRNAs was calculated using the comparative cycle threshold (Ct) method (^2-ΔΔ^Ct). Each data point was first normalized to U6 RNA as an internal control for inter-well variation or normalized to GAPDH as another internal control for inter-RNA sample variations. The final results are expressed as fold changes by normalizing the data to the values from non-AMI subjects.

**Animals**

C57BL/6 mice ranging from 10 weeks to 12 weeks in age and weighed between 25-30 g each were provided by the experimental animal Center of Harbin Medical University. Use of animals was in accordance with the regulations of the Ethic Committees of Harbin Medical University and conformed to the Guide for the Care and Use of Laboratory Animals published by the US National Institutes of Health (NIH Publication No. 85-23, revised 1996).

**Acute Myocardial Infarction Model (AMI)**

AMI was induced by left anterior descending coronary artery (LAD) ligation, as described in our previous study[3, 4]. In brief, mice were anesthetized by intraperitoneal injection of 60mg/kg sodium pentobarbital. The chest was opened to expose the heart. Under sterile conditions, an anterior transmural AMI was created by occlusion of the LAD with a 7-0 silk suture. Sham-operated mice, subject to an identical procedure with the suture being passed around the vessel without LAD occlusion, served as controls. Standard lead II ECG was recorded during and after 1 hour of the process of surgery.

**Collection and Handling of Mouse Blood Samples**

Blood samples were drawn directly from the hearts, and the hearts were then dissected post AMI 12h. These samples were immediately used for total RNA isolation as previously described [3].

**Statistical Analysis**

Categorical data were presented with count and percentile. Continuous variables were described as means ± SD, min, max, median and interquartile range. Student t test/Wilcoxon rank sum test was used to compare the demographic and clinical pathological characteristics between AMI patients and non-AMI subjects, and between AMI patients and healthy volunteers. Chi-squares were used to analyze the categorical data between two groups. Wilcoxon rank sum was performed to compare the expression of lncRNAs between AMI patients and non-AMI subjects, or between AMI patients and healthy volunteers. Univariable and multivariable logistic regression analyses were conducted to evaluate whether the lncRNAs are the independent factors for AMI. Univariate is used assuming that the response variable is influenced by only one other factor. Multivariate analysis is used to describe analyses of data where there are multiple variables or observations that may be are interrelated for each unit or individual. Thus, multivariate analysis considers the relationship between response variable and one other factor controlling for other variables. Generally, the multivariate analysis results are more reliable than the univariate ones. The area under Receiver–operator characteristic (ROC) curve (AUC) was used to evaluate the predictive power of circulating lncRNA levels for AMI. Spearman rank correlations were used to evaluate the association between levels of lncRNAs and cardiac risk factors, conventional AMI markers, and cardiac function parameters. All analyses were carried out with SAS 9.1 (Serial No. 989155) except that ROC was done with SPSS v17.0 software. The significant level was set at 0.05 and two-tailed P values <0.05 were considered statistically significant.

REFERENCES

[1] Alpert JS, Thygesen K, Antman E, Bassand JP. Myocardial infarction redefined--a consensus document of The Joint European Society of Cardiology/American College of Cardiology Committee for the redefinition of myocardial infarction. Journal of the American College of Cardiology 36, 959-69 (2000).

[2] Morrow DA, Cannon CP, Jesse RL, Newby LK, Ravkilde J, Storrow AB, et al. National Academy of Clinical Biochemistry Laboratory Medicine Practice Guidelines: Clinical characteristics and utilization of biochemical markers in acute coronary syndromes. Circulation 115, e356-75 (2007).

[3] Ai J, Zhang R, Li Y, Pu J, Lu Y, Jiao J, et al. Circulating microRNA-1 as a potential novel biomarker for acute myocardial infarction. Biochemical and biophysical research communications 391, 73-7 (2010).

[4] Yang B, Lin H, Xiao J, Lu Y, Luo X, Li B, et al. The muscle-specific microRNA miR-1 regulates cardiac arrhythmogenic potential by targeting GJA1 and KCNJ2. Nature medicine 13, 486-91 (2007).

**Table S1.** Human gene-specific primers for real-time PCR

| Gene name | Accession number | Chromosomal location | Forward primer (5’-3’) | Reverse primer (5’-3’) |
| --- | --- | --- | --- | --- |
| *ZFAS1* | NR_003604 | [hg19 chr20:47,894,715-47,905,797](http://genome.ucsc.edu/cgi-bin/hgTracks?db=hg19&position=chr20:47,894,715-47,905,797) | AACCAGGCTTTGATTGAACC | ATTCCATCGCCAGTTTCT |
| *CDR1AS* | NC_000023.10 | [hg19 chrX:139,865,328-139,866,829](http://genome.ucsc.edu/cgi-bin/hgTracks?db=hg19&position=chrX:139,865,328-139,866,829) | TCTGCTCGTCTTCCAACATC | AGATCAGCACACTGGAGACG |
| *SRA* | NR_045586 | [hg19 chr5:139,929,652-139,937,678](http://genome.ucsc.edu/cgi-bin/hgTracks?db=hg19&position=chr5:139,929,652-139,937,678) | GGAAGCAGGTATGTGATGAC | TACCATCCACTGACTGACCT |
| *SAF* | NR_028371.1 | [hg19 chr10:90,751,182-90,752,732](http://genome.ucsc.edu/cgi-bin/hgTracks?db=hg19&position=chr10:90,751,182-90,752,732) | ACATCTCAGCCTCTTGGTG | ACAGATGGCGAAATGAGG |
| *NESPAS* | NR_002785.2 | [hg19 chr20:57,393,973-57,425,958](http://genome.ucsc.edu/cgi-bin/hgTracks?db=hg19&position=chr20:57,393,973-57,425,958) | GGGAAGACCACAAAAGCAT | GATGACCCAGCACAAAAAC |
| *MIAT* | NR_003491.2 | [hg19 chr22:27,042,392-27,072,440](http://genome.ucsc.edu/cgi-bin/hgTracks?db=hg19&position=chr22:27,042,392-27,072,440) | GAACCCAGAGACATGATCCC | AAAGAGAAGTCCAGGCCAGG |
| *NRON* | NR_045006.1 | [hg19 chr9:129170054-129172783](http://genome.ucsc.edu/cgi-bin/hgTracks?db=hg19&position=chr9:129170054-129172783) | GACCAATGCAACTCCAACCT | CATCTTCCAGTGGCAGCTTT |
| *ANRIL* | NR_003529.3 | [hg19 chr9:21,994,790-22,121,093](http://genome.ucsc.edu/cgi-bin/hgTracks?db=hg19&position=chr9:21,994,790-22,121,093) | TCTCATTGGGGATACGAAGC | GGATCACAGACCATACTTGC |
| *DIO3OS* | NR_002770.1 | [hg19 chr14:102,018,560-102,026,740](http://genome.ucsc.edu/cgi-bin/hgTracks?db=hg19&position=chr14:102,018,560-102,026,740) | CTTCCTGCTCTTCGTTGTCC | TGAGGAGGATTGAGTTGGG |
| *SENCER* | NR_038908 | [chr11:128561567-128565918](http://genome.ucsc.edu/cgi-bin/hgTracks?hgsid=409833575_zu0YNnndYpba41HXI65CntVBkqYr&db=hg19&position=chr11:128561567-128565918) | TCAGAAGAGGCCTTCAGAGC | CTCGAGAAGATGCGGATAGG |
| *FENDRR* | NR_036444 | [chr16:86521271-86542466](http://genome.ucsc.edu/cgi-bin/hgTracks?hgsid=409833575_zu0YNnndYpba41HXI65CntVBkqYr&db=hg19&position=chr16:86521271-86542466) | AATTGCTGGGCTGCTTTCTA | TTCACAATGGCTCAGTGCTC |
| *aHIF* | HSU85044 | [chr16:86521271-86542466](http://genome.ucsc.edu/cgi-bin/hgTracks?hgsid=409833575_zu0YNnndYpba41HXI65CntVBkqYr&db=hg19&position=chr16:86521271-86542466) | TTTGTGTTTGAGCATTTTAATAGG C | CCAGGCCCCTTTGATCAGCTT |
| *HCG22* | NR_003948 | [chr6_ssto_hap7:2356208-2362626](http://genome.ucsc.edu/cgi-bin/hgTracks?hgsid=409833575_zu0YNnndYpba41HXI65CntVBkqYr&db=hg19&position=chr6_ssto_hap7:2356208-2362626) | CGCAGGCACAAATGGATGAG | CTGGTCTCTTTCCGTGGGAC |
| *MHRT* | NR_126491 | [chr14:23884659-23886804](http://genome.ucsc.edu/cgi-bin/hgTracks?hgsid=409833575_zu0YNnndYpba41HXI65CntVBkqYr&db=hg19&position=chr14:23884659-23886804) | CCGACTGCGACTCCTCATAC | GGCTGAAGAGTGAGCCTTGT |
| *CARL* | JX003871 | [chr8:128197725-128200455](http://genome.ucsc.edu/cgi-bin/hgTracks?hgsid=409833575_zu0YNnndYpba41HXI65CntVBkqYr&db=hg19&position=chr8:128197725-128200455) | GACTGACTGTGATGGCACCC | TGGGATGCCTCAACACAAGA |
| GAPDH |  |  | GGTGAAGCAGGCGTCGGAGG | GAGGGCAATGCCAGCCCCAG |
| miR-1 |  |  | TGGAATGTAAAGAAGT | CAGTGCGTGTCGTGGAGT |
| U6 |  |  | GCTTCGGCAGCACATATACTAAAAT | CGCTTCACGAATTTGCGTGTCAT |

Mouse gene-specific primers for real-time PCR

| Gene name | Accession number | Chromosomal location | Forward primer (5’-3’) | Reverse primer (5’-3’) |
| --- | --- | --- | --- | --- |
| *ZFAS1* | NM_001081005.1 | [mm9 chr2:166,888,434-166,891,362](http://genome.ucsc.edu/cgi-bin/hgTracks?db=mm9&position=chr2:166,888,434-166,891,362) | AGCGTTTGCTTTGTTCCC | CTCCCTCGATGCCCTTCT |
| *CDR1AS* | NC_000086.6 | [mm10 chrX:61,183,236-61,186,179](http://genome.ucsc.edu/cgi-bin/hgTracks?db=mm10&position=chrX:61,183,236-61,186,179) | TCTATGCCTTCCACAAATCC | CGAAGACATGGATTTTGGG |
| GAPDH |  |  | GGTGAAGCAGGCGTCGGAGG | GAGGGCAATGCCAGCCCCAG |
